# Image Super Resolution-Based Channel Estimation for Orthogonal Chirp Division Multiplexing on Shallow Water Underwater Acoustic Communications

**DOI:** 10.3390/s24092846

**Published:** 2024-04-29

**Authors:** Haoyang Liu, Chuanlin He, Yanting Yu, Yiqi Bai, Yufei Han

**Affiliations:** 1 School of Ocean Technology Sciences, Qilu University of Technology (Shandong Academy of Science), Qingdao 266100, China; lhy726208@163.com; 2 School of Ocean Engineering, Harbin Institute of Technology (Weihai), Weihai 264209, China; 3 Institute of Oceanographic Instrumentation, Qilu University of Technology (Shandong Academy of Science), Qingdao 266100, China; 4 Tangshan Institute of Southwest Jiaotong University, Southwest Jiaotong University, Tangshan 063000, China

**Keywords:** deep learning-based channel estimation, orthogonal chirp division multiplexing, underwater acoustic communication

## Abstract

Orthogonal chirp division multiplexing (OCDM) offers a promising modulation technology for shallow water underwater acoustic (UWA) communication systems due to multipath fading resistance and Doppler resistance. To handle the various channel distortions and interferences, obtaining accurate channel state information is vital for robust and efficient shallow water UWA communication. In recent years, deep learning has attracted widespread attention in the communication field, providing a new way to improve the performance of physical layer communication systems. In this paper, the pilot-based channel estimation is transformed into a matrix completion problem, which is mathematically equivalent to the image super-resolution problem arising in the field of image processing. Simulation results show that the deep learning-based method can improve the channel distortion, outperforming the equalization performed by traditional estimator, the performance of Bit Error Rate is improved by 2.5 dB compared to the MMSE method in OCDM system. At the 7.5 to 20 dB region, it achieves better bit error rate performance than OFDM systems, and the bit error rate is reduced by approximately 53% compared to OFDM when the SNR value is 20, which is very useful in shallow water UWA channels with multipath extension and severe time-varying characteristics.

## 1. Introduction

Underwater acoustic (UWA) channels are widely considered one of the most demanding communication mediums [1,2,3], especially in shallow water UWA channels with severe multipath effects and Doppler frequency shifts. In contrast to terrestrial communication situations, the characteristics of the shallow water UWA channel are affected by diverse environmental factors, including multipath effect, Doppler frequency shift, transmission loss, ambient ocean noise, limited bandwidth, etc. [4]. Compared to underwater optical wireless communication, UWA communication features lower data rates, narrower bandwidth, higher propagation delay, and weaker security [5]. However, the absorption and scattering of optical signals in water severely constrain communication distances. Moreover, factors such as bubbles, turbulence, carrier jitter, and underwater obstacles in shallow sea environments diminish the reliability of transceivers. UWA communication has become the most widely used underwater wireless communication method due to its relative robustness in long-distance and complex environments. Orthogonal frequency division multiplexing (OFDM) has been applied in UWA communication for resisting the resistance to inter symbol interference (ISI), high spectrum utilization, anti-multipath fading, and high-speed transmission capabilities. Now, it has become the most commonly used multi-carrier scheme for UWA communication [6]. However, the existence of Doppler frequency shift in the shallow water UWA channel destroys the orthogonality of OFDM subcarriers, which greatly reduces the performance of OFDM in dual-selection channels. This undoubtedly increases the complexity of the OFDM system.

Since the autocorrelation function of the Chirp signal offers precise time resolution and is more insensitive to the Doppler shifts [7], it holds a significant advantage in addressing Doppler shifts within the shallow water acoustic channel. The difference between OFDM and orthogonal chirp division multiplexing (OCDM) are as follows: OFDM is based on the Fourier transform, which consists of a set of orthogonal subcarriers that are modulated independently, the subcarrier frequency does not vary with time and is sensitive to Doppler shifts, the orthogonality of subcarriers is disrupted in shallow water acoustic channels, leading to performance degradation. OCDM is based on the Fresnel transform [8], which consists of multiple orthogonal chirp waveforms sharing the same bandwidth and time slot. OCDM signals enhance data transmission reliability by effectively utilizing multipath diversity and exhibiting stronger fading resistance when compared to OFDM signals [9]. Similar to the OFDM communication system, channel estimation is crucial in the UWA–OCDM communication system. OCDM signals will be distorted when passing through a shallow water acoustic channel (SWAC), an accurate channel state information (CSI) is required to compensate the distortion from the channel and to restore the transmission signal. Pilot-based channel estimation is the most widely used method of obtaining CSI, as both data symbols and pilot symbols are sent together for channel estimation. Traditional frequency–domain equalizers used in OCDM include the zero forcing (ZF) equalizer and the minimum mean square error (MMSE) equalizer. Among them, the ZF equalizer does not use prior information and is extremely sensitive to noise; therefore, its performance at low signal-to-noise ratios (SNR) is often unsatisfactory. The MMSE equalizer can effectively balance the noise and compensate the channel, making its performance better than the ZF equalizer [10].

Deep learning, an artificial intelligence technique, excels in addressing complex nonlinear problems and performs well in fields like computer vision and natural language processing [11]. It has seen a substantial rise in its application within the realm of communication, providing a new avenue for enhancing the performance of physical layer communication systems [12,13]. Deep learning has proven effective in OFDM communication scenarios where the channel is either unknown or of a high complexity [14,15,16]. In [17], a neural network is trained to fit the transmission process of the channel, and the receiver of OFDM mainly learns how to recover the transmitted symbols from the received signal, rather than estimating the time-frequency response of the channel. In [18], an OFDM communication receiver system based on a five-layer fully connected deep neural network was developed for channel estimation and equalization in UWA communication. The model performed advantages over traditional algorithms in situations with limited pilot subcarriers. In [19], a computer vision tool is applied in channel estimation work for the first time, the authors estimate the channel matrix with the help of a denoising convolutional neural network (DnCNN). In [20], the channel response is regarded as an image, meaning that image super-resolution (SR) and image restoration (IR) techniques are introduced. The specific method is cascaded super-resolution convolutional neural network (SRCNN) and denoising neural network (DnCNN) completes channel estimation, and its performance is approximately equal to the MMSE estimator at low SNR. In [21], the up-sampling function in the network is implemented as transposed convolution layer, which can scale up image height and width by different factors, which is suitable for any pilot pattern.

Considering that the use of a deep learning-based channel estimation method can achieve a certain performance improvement compared to traditional channel estimation methods, it can achieve higher interpolation accuracy and noise reduction. Very deep super-resolution (VDSR) is one of the most representative networks in the field of deep convolutional neural networks for super-resolution, which can be utilized as the foundation for designing channel estimation neural network. This paper proposes an OCDM channel estimation method based on SR technology which improves the performance and capability of multicarrier underwater acoustics communication systems in shallow water acoustic channels.

The remainder of this paper is organized as follows: in Section 2, a brief survey of the OCDM model as well as the relationship between channel estimation and super-resolution problem are formulated. Section 3 presents the structure of the super resolution-based channel estimation method. The simulation results are presented in Section 4, and the conclusions are summarized in Section 5.

## 2. Related Works and Problem Formulation

### 2.1. OCDM System Model

**Figure 1:**
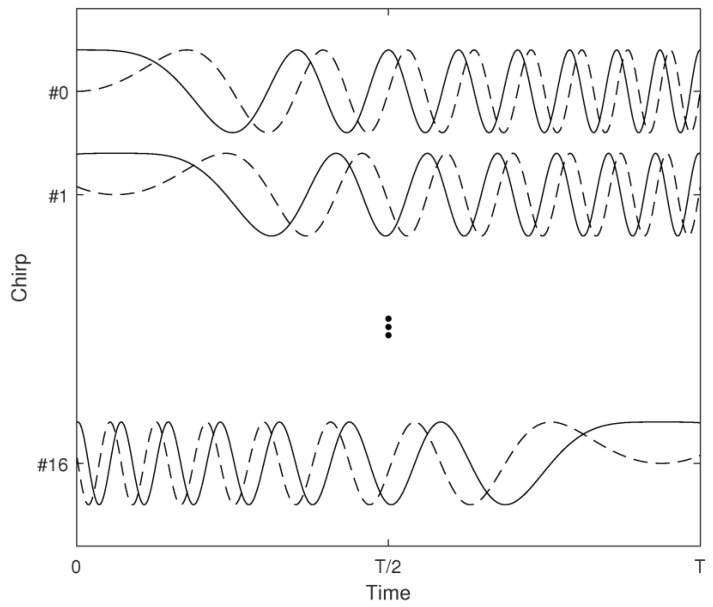
OCDM chirp waveforms for N.

The OCDM system model is shown in Figure 1. The bitstream data is transformed from serial to parallel at the transmitter, followed by encoding and mapping. Equation (1) shows the OCDM time domain signal is obtained through inverse discrete Fresnel transform (IDFnT) from multiplexing N orthogonal chirp waves.

(1) $$s\left(n\right)=\sum_{k=0}^{N-1}x\left(k\right)\psi_{k}\left(n\frac{T}{N}\right)=e^{j\frac{\pi }{4}}\sum_{k=0}^{N-1}x\left(k\right)e^{-j\frac{\pi }{N}{\left(n-k\right)}^{2}}.$$

The chirp waves as shown in Figure 1. Solid lines represent in-phase components and dashed lines represent orthogonal components.

Transform above expression in a concise matrix form, shown in Equation (2).

(2) $$s=\Phi^{H}x.$$

In Equation (2), $x=\left[x\left(0\right),x\left(1\right),\ldots ,x\left(N-1\right)\right]$ and DFnT unitary matrix $\Phi \in C^{N*N}$ is defined as Equation (3),

(3) $${\left\{\Phi \right\}}_{m,n}=\frac{1}{\sqrt{N}}e^{-j\frac{\pi }{4}}exp\left(j\frac{\pi }{N}{\left(m-n\right)}^{2}\right).$$

After IDFnT, N parallel subcarriers are converted into post-serial bit streams. After that, a cyclic prefix sequence is added at the head of data streams to avoid ISI. The transmitting signal after the UWA channel can be concluded as Equation (4).

(4) $$r=Hs+n=H\Phi^{H}x+w$$

where w is the Gaussian white noise vector in the channel, the circulant matrix H is the channel impulse response (CIR), and the first column is:

(5) $$h=\left[h\left(0\right),h\left(1\right),\ldots ,h\left(L-1\right),0,\ldots ,0\right].$$

where $h\left(l\right),l=0,\ldots ,L-1$ is the first moment’s CIR tap and L is the maximum delay spread of the channel.

To reduce computational load, based on the eigen-decomposition properties of the DFnT matrix [22], the discrete FFT transform is employed for DFnT implementation, which is shown in Equation (6).

(6) $$y=F\cdot r=\Gamma^{H}\Lambda Fx+w.$$

where F is a Fourier matrix of size N, and **Γ** and Λ are both diagonal matrices of size N∗N. The k-th diagonal term of Λ is the k-th frequency point of channel frequency response (CFR). When the size of N is even: $\Gamma_{n,n}=e^{-j\frac{\pi }{N}n^{2}}$.

Therefore, both ZF and MMSE channel equalization matrix G in the above expressions can be obtained from Equations (7) and (8):

(7) $$G_{ZF}=\Lambda^{H}/\Lambda^{H}\Lambda .$$

(8) $$G_{MMSE}{=\Lambda }^{H}/(\Lambda^{H}\Lambda +SNR^{-1})$$

**Figure 2:**
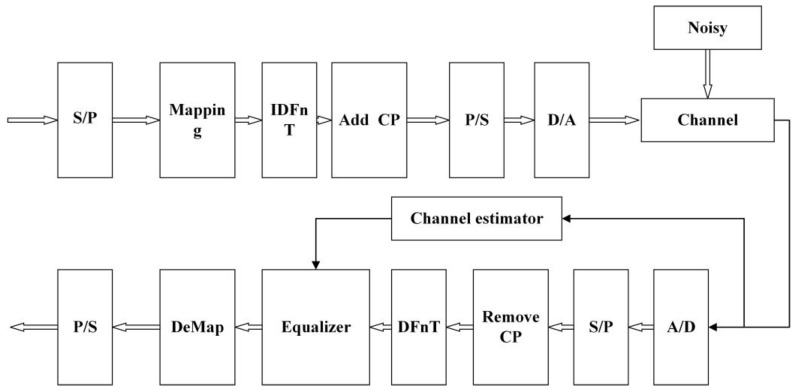
Structure of OCDM system in UWA channel.

The OCDM system model as shown in Figure 2, the transmitter transforms bitstream data from serial to parallel and generally utilizes modulation to achieve higher data transmission rates. Each modulated subcarrier can carry more information, thereby achieving higher spectral efficiency. A discrete time–domain signal is obtained by IDFnT. Due to inter-symbol interference caused by multipath effect, additional guard intervals are inserted into adjacent blocks. Typically, cyclic prefixes are used to fill guard intervals. The length of the guard interval depends on the specific channel environment, it should be greater than the maximum delay of the channel. After serial-to-parallel conversion, the UWA transducer only needs to convert the digital signal into its analog counterpart for transmission through seawater. Upon successful synchronization at the receiver, the subsequent procedure entails the digitization of the captured signal through quantization and sampling. After removing the cyclic prefix from the signal, DFnT operations are applied to demodulate data from each subcarrier. Due to channel interference, the receiver conducts channel estimation to obtain accurate channel information to recover the signal.

### 2.2. Single Image Super-Resolution and Channel Estimation

**Figure 3:**
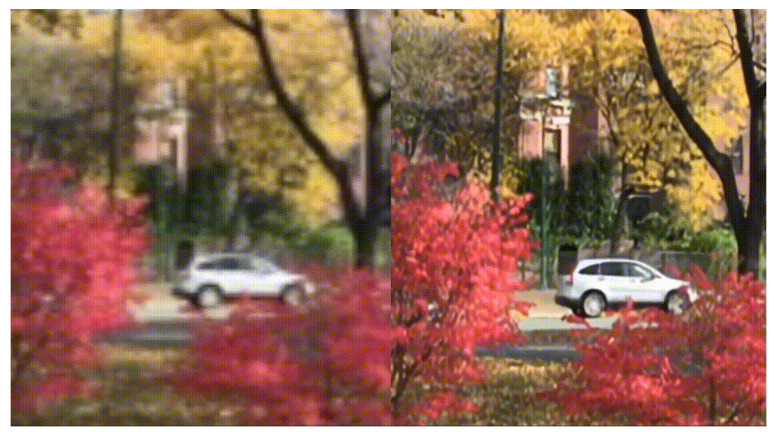
Upgrade of low-resolution image to high-resolution image.

Single image super-resolution is an image processing algorithm in the field of computer vision which aims to upgrade a low-resolution image to a high-resolution image, the result of single image super-resolution is shown in Figure 3. The mathematical model of single image super-resolution can be seen as a matrix imputation problem, using known data point value to infer surrounding unknown data point value.

**Figure 4:**
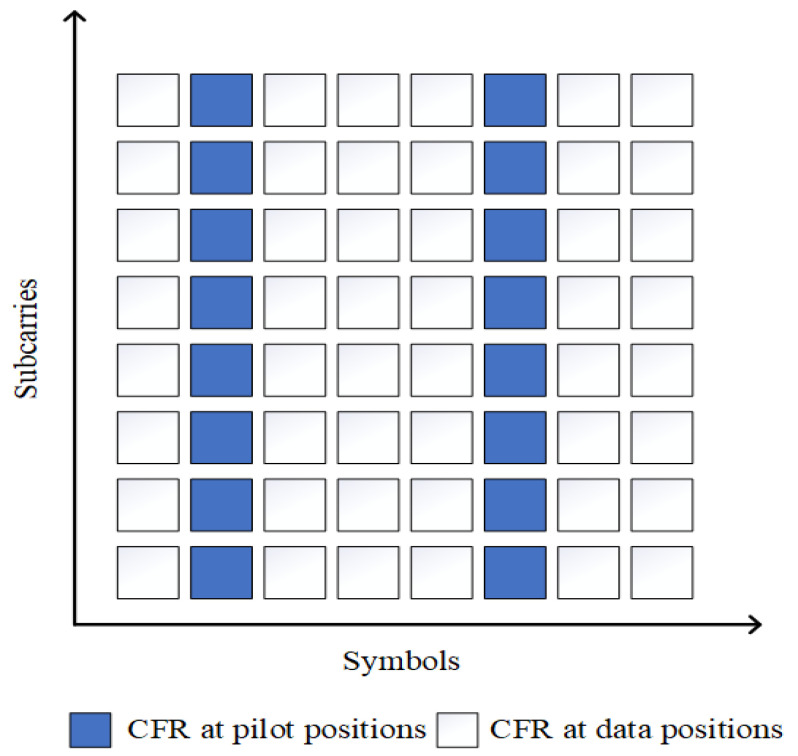
Principle of channel estimation assisted by pilot sequences.

As shown in Figure 4, in channel estimation assisted by pilots, the CFR at the pilot position is pre-estimated, which is the known data point value in single image super resolution situation. However, the CFR at the data position is unknown and needs to be inferred using the CFR obtained from pilot positions, which is the surrounding unknown data point value in single image super resolution situation. The time-frequency grid of the SISO communication link channel response is modeled as an image known only at the pilot locations. Consider the channel response in the pilot positions as a low-resolution image and the estimated channel response as the proposed high-resolution image. In the process of promoting resolution, the whole time-frequency response channel is recovered by the transmitted pilot. Given that pilot-aided channel estimation has a similar problem formulation with image super-resolution, it is reasonable to introduce computer vision tools to solve the problem of underwater acoustic channel estimation.

The image super-resolution mathematical expression is shown by Equation (9):

(9) $$L_{h}=f\left(L_{l};\theta \right)$$

where $L_{l}$ is the blurry low-resolution image, $L_{h}$ is the restored high-resolution image, f is the super-resolution model, and its parameter is denoted as θ.

Similarly, the relationship between estimated CFR and genius CFR can be concluded as Equation (10):

(10) $$\hat{H}=f\left(\hat{H_{p}};\theta \right)$$

where $\hat{H_{p}}$ represents the CFR at the pilot within a transmission frame and $\hat{H}$ represents the complete CFR of the transmission frame.

## 3. Proposed Methods

### 3.1. Network Architecture

The VDSR network model consists of 20 layers, each of which is followed by the PReLu activation function, enabling VDSR to possess a wide receptive field, thereby further leveraging contextual information effectively. The first layer is the input layer, which is a convolutional layer with 64 filters of size 3 × 3 × 2, and the last layer is the output layer, which has 2 filters of size 3 × 3 × 64. The remaining layers are hidden layers, and each of them is composed of convolutional layers with filters of size 3 × 3 × 64. The result of last convolutional layer is residual, the output is the sum of residual and input.

**Figure 5:**
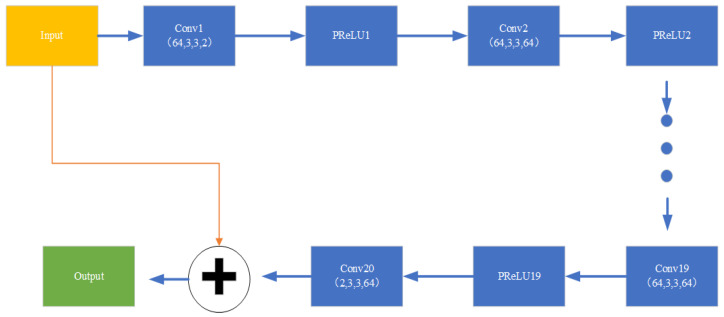
Architecture of VDSR for channel estimation.

As shown in Figure 5, the weight factors between adjacent convolutional layers are dynamically adjusted through the backpropagation algorithm during the weight training process. Adjacent convolutional layers are connected by the parametric rectified linear unit (PReLU) activation function.

**Figure 6:**
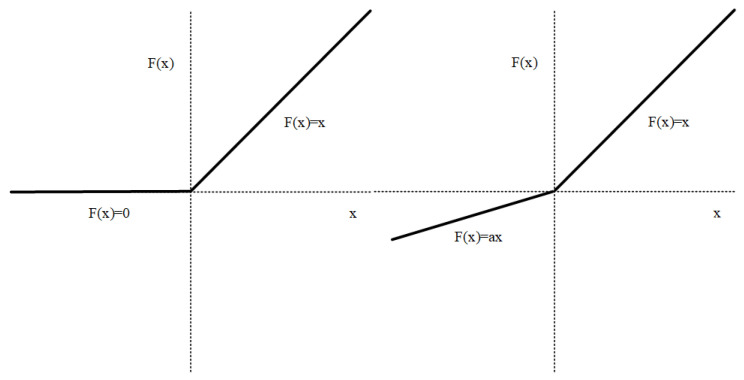
ReLU function (**left**) and PReLu function (**right**).

As shown in Figure 6, the ReLU function outputs zero continuously for negative inputs whereas PReLU allows the introduction of learnable parameters for negative inputs, enabling the output to be non-zero. As shown in Equation (11), a small slope a is introduced in the negative input interval, the problem of gradient disappearance caused by negative numbers is alleviated and the problem of neurons not learning due to negative numbers is prevented. The difference between LReLU and PReLU resides in the fact that the slope of LReLU requires manual setting, while the slope of PReLU serves as a learned parameter and does not require manual setting.

(11) $$PReLU\left(x\right)=\left\{\begin{array}{c} x\;\;\;if\;x>0\\ ax\;\;\;\;\;\;\;\;\;else \end{array}\right.$$

### 3.2. Procedure of the Method

In order to diminish the overall complexity, the receiver uses least squares (LS) estimation to obtain the CFR based on the OFDM signal at the pilot position. The LS estimation fails to consider the influence of noise without using prior information, leading to a certain level of discrepancy with the actual CFR. Since the data is unknown except for the pilot position, the upsampling operation is required when the CFR matrix is sent to the neural network. Specifically, the nearest neighbor interpolation is used to initially estimate the frequency domain response at the data position with the help of the pilot position.

**Figure 7:**
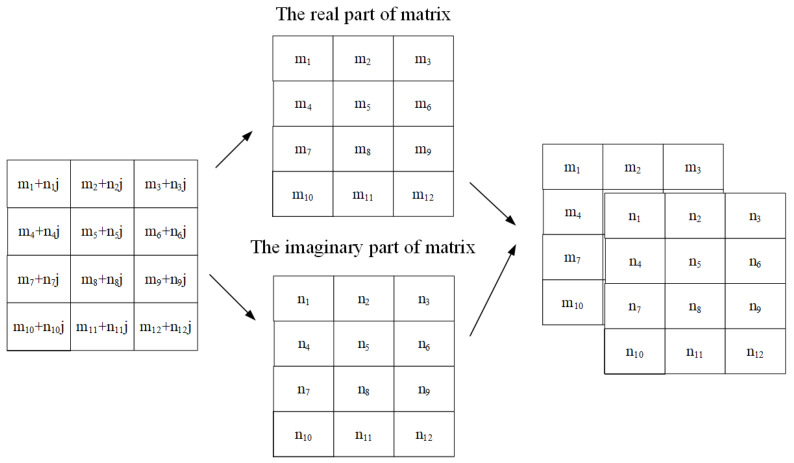
Convert complex channel matrix to two-channel real matrix.

There is one more thing that needs to be dealt with before model training: the matrices processed by the VDSR network are real values, whereas the CFR matrix is a complex matrix. Inspired by the processing of multi-channel color images by VDSR, the complex matrix is converted into a two-channel real matrix, as shown in Figure 7. After training, the output CFR matrix completes high-precision interpolation and noise reduction at the data positions, which is the prediction of the real CFR matrix.

**Figure 8:**
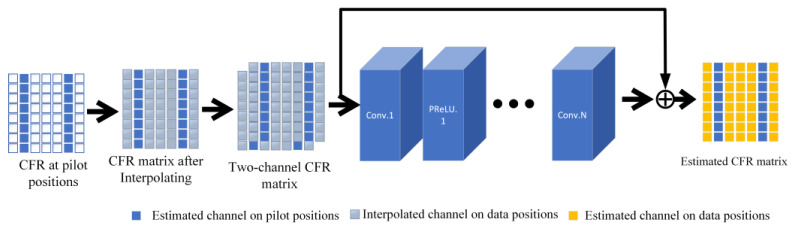
Overall process of using VDSR method for UWA channel estimation.

The super resolution-based channel estimation method is illustrated in Figure 8.

### 3.3. Model Training

As shown in Equation (12), VDSR employs the L2 loss function for supervised learning and training.

(12) $$Loss\left(\theta \right)={\left\|\hat{H}-H\right\|}_{2}$$

where $\hat{H}$ is estimated CFR and H is real CFR.

In the process of model training, the values of the CFR matrix data are very small, which make them difficult to train. Therefore, an additional procedure is required to enlarge the data of the CFR matrix to a certain extent, making the data magnitude range between 10^−1^ and 10^−3^. In the early stages of training, the model uses a larger learning rate to quickly approach the global optimal solution area while avoiding falling into the local optimal solution. When approaching the optimal solution, the learning rate gradually decreases to make the model converge to the minimum value in smaller steps. At the same time, the optimizer uses stochastic gradient descent (SGD) with momentum, which leads the parameter update smoother and has inertia, thereby speeding up the convergence speed, enhancing the stability of the training process and the generalization ability of the model.

## 4. Experiment Results

**Figure 9:**
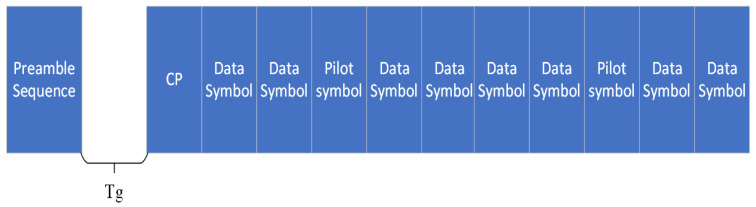
Frame structure.

In this section, in order to test the performance of VDSR in channel estimation, BER and MSE are used as reference indicators to compare with traditional channel estimation methods of OCDM and OFDM systems. The frame structure of OFDM and OCDM are shown in Figure 9, the purpose of a preamble sequence in signal transmission is to facilitate synchronization and timing at the receiver, and $T_{g}$ is the time interval between the preamble sequence and the data.

### 4.1. Experiment Parameter Settings

In this experiment, Doppler frequency shifts disrupt the orthogonality of subcarriers in shallow water acoustic channels, and OCDM performs well in shallow water acoustic channels due to its insensitivity to Doppler frequency shifts. However, in deep water acoustic channels where there is no significant Doppler spread, so using OCDM offers no advantage and may instead increase complexity. Therefore, the application scenario is targeted towards shallow water acoustic channels with significant Doppler spread. Shallow water acoustic channel usually has a depth below 100 m and the length from 100 m to 3000 m. The parameters of the simulation are listed in Table 1. The pilot is OFDM signal and uses block-type pilots. The simulator considers the influence of environmental factors such as sound velocity profile, sea surface, and seabed depth.

**Figure 10:**
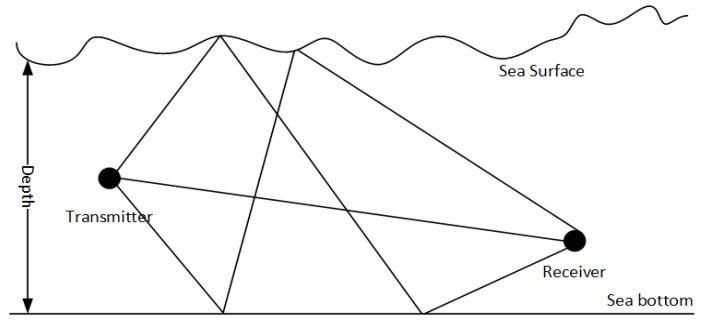
Model of shallow UWA multipath channel.

As shown in Figure 10, a SISO communication scheme is applied in the simulation. Mathematical representation of the UWA multipath channel is shown as Equation (13),

(13) $$h\left(t\right)=\sum_{i=0}^{m-1}h_{i}\delta \left(t-\tau_{i}\right),$$

where $h\left(t\right)$ is impulse response function of the channel, m is the total number of multipaths, $h_{i}$ is discrete complex gain, and $\delta \left(t-\tau_{i}\right)$ is impulse response delayed by $\tau_{i}$. The depth of the sea is 100 m. The transmitter is positioned 50 m above the sea bottom, the height of the receiver from the bottom is 30 m, and the distance between the two is 300 m, 800 m, and 3000 m.

**Figure 11:**
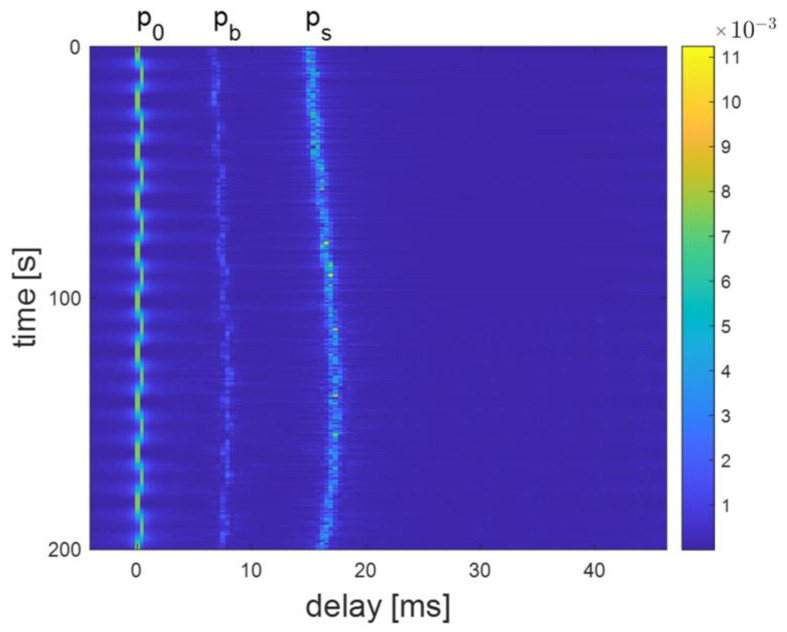
Delay plot of channel modem at 300 m distance.

**Figure 12:**
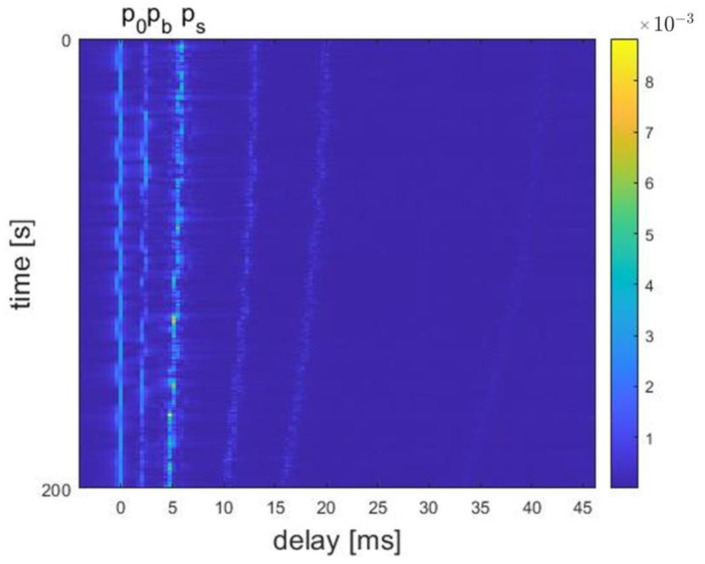
Delay plot of channel model at 800 m distance.

**Figure 13:**
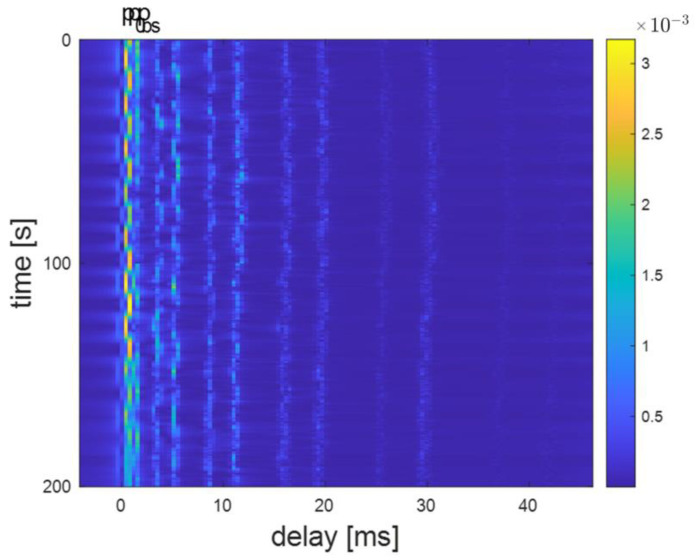
Delay plot of channel model at 3000 m distance.

The channel delay plots at distances of 300 m, 800 m, and 3000 m are shown in Figure 11, Figure 12, and Figure 13, respectively. For example, Figure 12 shows a clear multipath structure of the channel, primarily manifested in the five arrival peaks of the signal. The channel remains overall stable with slow temporal variations, consistent with the characteristics of shallow water channels. The number of arrival peaks at 300 m is fewer than at 800 m, while the number of arrival peaks at 3000 m is greater than at 800 m.

**Figure 14:**
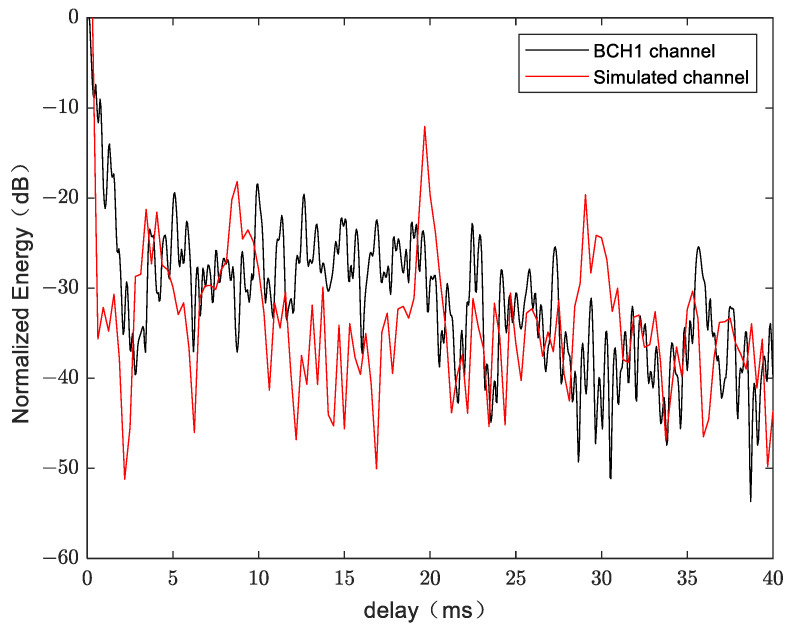
Normalized channel impulse response.

The normalized energy from both BCH1 and simulation at a distance of 800 m are shown in Figure 14. BCH1 is a SIMO channel from the commercial harbor of Brest, France. A single probe transmission over a range of 800 m resulted in a 59.4 s channel estimate, simultaneously recorded on the receive hydrophones [23]. In Figure 14, it can be seen that the simulation channel shares the same multipath features with real shallow water acoustic channel.

In the simulation, 20,000 CFR matrices are generated for each channel model, which are randomly split into three subsets: 80% are designated as the training set, 10% as the validation set, and the final 10% are reserved for the test set. The size of the channel matrix is 128 × 10—128 corresponds to the number of subcarriers, 10 represents the number of symbols per frame—and the third and seventh symbols are used as pilot symbols of the frame. In addition, the initial learning rate is 0.005, and then the learning rate decays to the previous 0.1 every 20 rounds, and the maximum number of training rounds is 100. To mitigate the risk of overfitting, the training process will utilize early stopping, halting if the value of the loss function fails to decrease further over five consecutive epochs. This strategy helps prevent the network from overfitting by discontinuing the learning process once it has captured the essential statistical properties of the training data, thereby avoiding unnecessary refinement on individual features.

In actual UWA communication application scenarios, the SNR tends to fluctuate with time, so it is not feasible to train a weight model under a single SNR. In order to make the trained network applicable to a variety of application scenarios, the neural network is trained by a series of signal-to-noise ratio data instead of data under a single SNR. Therefore, channel samples of [0–20] dB are mixed in the training process dataset.

### 4.2. Mean Square Error

In the experiment, a number of different equalization methods are investigated to evaluate the performance of the proposed VDSR channel estimation system. OCDM MMSE and OCDM ZF represent channel equalization using the channel state information obtained by MMSE and ZF channel estimation algorithms for OCDM communications, respectively. Similarly, OFDM LMMSE and OFDM LS represent channel equalization using the channel state information obtained by LMMSE and LS channel estimation algorithms for OFDM communications, respectively. OFDM DL and OCDM DL represent the equalization of the channel estimated by the enhanced super resolution model VDSR for OFDM and OCDM, respectively.

**Figure 15:**
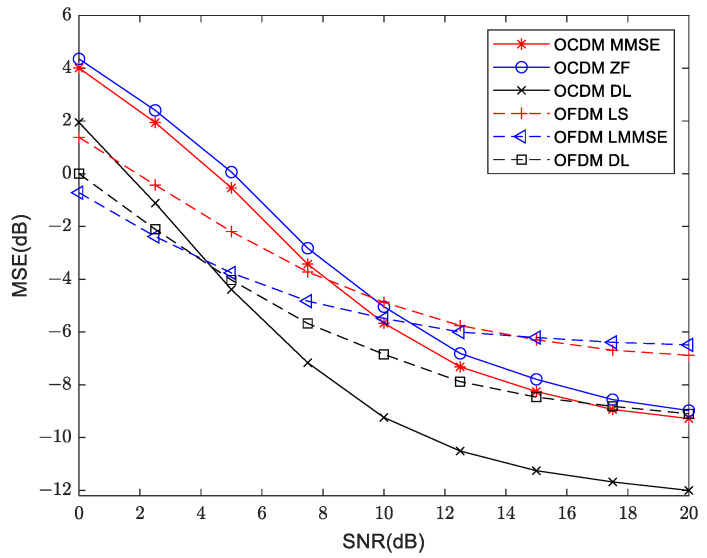
Mean square error measurement in traditional and deep learning approaches for OFDM and OCDM at 800 m distance.

As shown in Figure 15, the trends in OFDM and OCDM in the shallow water acoustic channel at a distance of 800 m are similar, where MSE decreases with SNR increases. The lower bound of MSE to OFDM is −6 dB whereas OCDM is −8 dB. In high SNR regions OCDM outperforms OFDM, which is consistent with the theoretical predictions. However, when SNR is below 7.5 dB (i.e., low SNR region), the MSE of OCDM is higher than OFDM due to a frequency point estimation error whereby the OCDM modulation scheme based on Chirp subcarriers results in data detection errors across all subcarriers, whereas OFDM only incurs detection errors at specific data points. By applying deep learning, it is observed that MSE is significantly reduced in both OFDM and OCDM cases. In OFDM cases, it can be reduced to −8 dB. Particularly noteworthy is the case of OCDM, where it can be reduced to −12 dB. The combination of OCDM and VSDR can reduce the MSE from −8 dB to −12 dB. This indicates that the enhanced VDSR model -based channel estimation neural network trained by joint SNR training exhibits high versatility, enabling it to handle noise of varying intensities.

**Figure 16:**
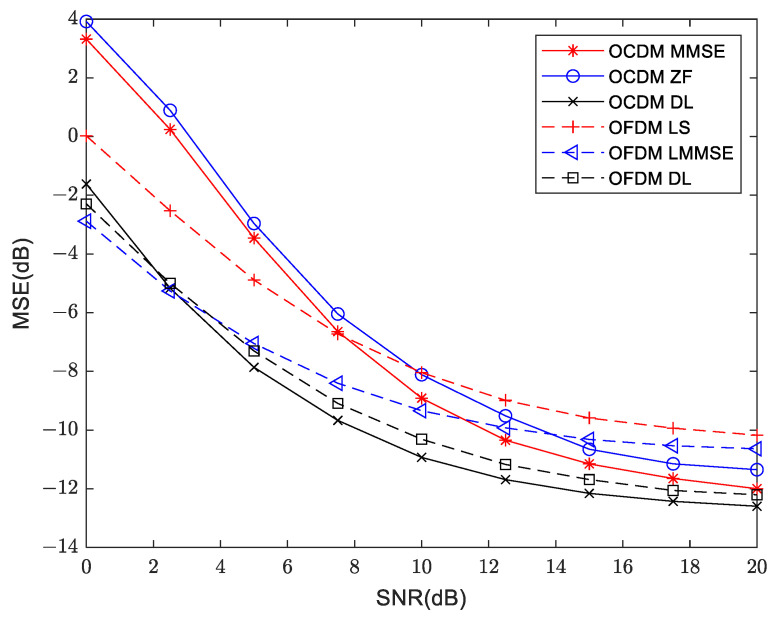
Mean square error measurement in traditional and deep learning approaches for OFDM and OCDM at 300 m distance.

**Figure 17:**
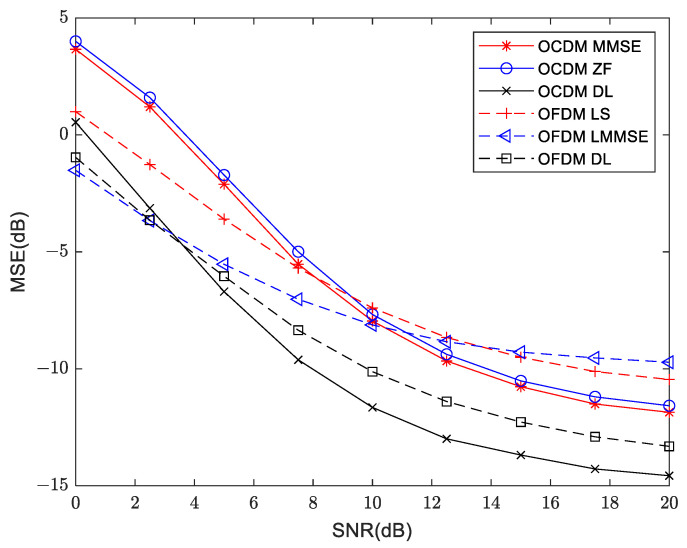
Mean square error measurement in traditional and deep learning approaches for OFDM and OCDM at 3000 m distance.

The mean square error measurement at 300 m and 3000 m are shown in Figure 16 and Figure 17, respectively. It can be seen from the three sets of channel models that the lower limit of MSE for OCDM is lower than that for the OFDM system, and using deep learning can reduce the lower limit of MSE for both OFDM and OCDM.

### 4.3. Bit Error Rate

**Figure 18:**
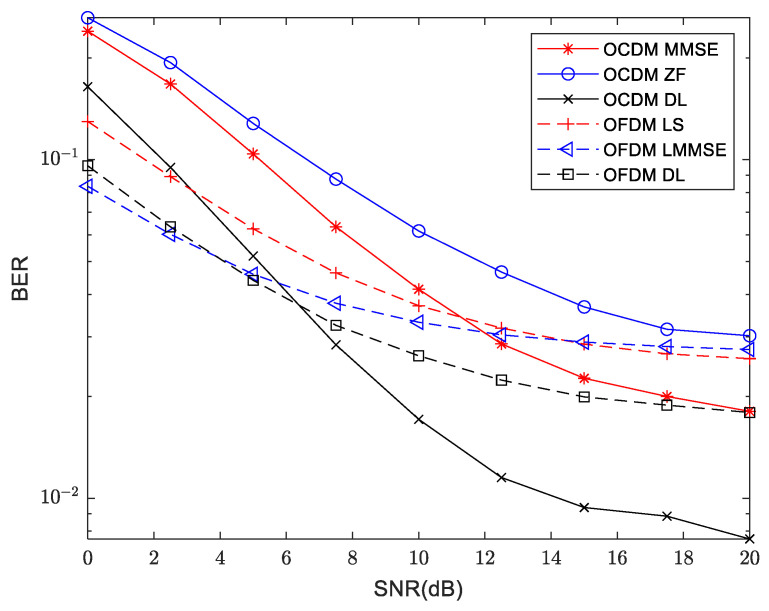
Comparison of BER between traditional and deep learning approaches for OFDM and OCDM at 800 m distance.

The bit error rate (BER) performance of OFDM and OCDM in the 800 m shallow water acoustic channel is shown in Figure 18. OCDM shows a higher BER within the 0–10 dB SNR range in contrast to OFDM, indicating inferior performance. However, as SNR increases, OCDM’s performance starts to surpass that of OFDM LS. Furthermore, at an SNR of 12.5 dB, OCDM MMSE has already outperformed OFDM LMMSE, which is obvious at higher SNRs. For instance, the BER has been reduced by 35.1% and 36.5% at 15 dB and 20 dB, respectively, compared to OFDM LMMSE. OCDM equalization causes a series of data determination errors due to a frequency point estimation error. Therefore, the performance of OCDM is not as good as OFDM under the low SNR region, but the performance of OCDM has surpassed OFDM under medium and high SNR. Comparing the deep learning method with traditional methods, for OFDM DL equalization, the signal experiences a slightly higher BER than OFDM LMMSE within the 0 to 2.5 dB SNR range. Specifically, the BER of OFDM DL is approximately 15.1% higher than that of OFDM LMMSE method at 0 dB. However, beyond this range, OFDM DL consistently outperforms OFDM LMMSE at 2.5–20 dB. Moreover, at 20 dB, OFDM DL attains a performance level that is on par with that of OCDM MMSE. In the case of OCDM DL equalization, the signal performs better across the entire SNR range when compared to traditional ZF and MMSE methods. Due to frequency point estimation error leading to a series of chirp determination errors, the BER performance of OCDM DL is inferior to that of OFDM LMMSE and OFDM DL methods in the low SNR range of 0–5 dB, but is superior to the OFDM LS after 2.5 dB. When the SNR is higher than 7.5 dB, the improvement of OCDM DL is remarkable, which is the best among the six equalization methods, the BER has been reduced by approximately 53% compared to the OFDM DL method at 20 dB.

**Figure 19:**
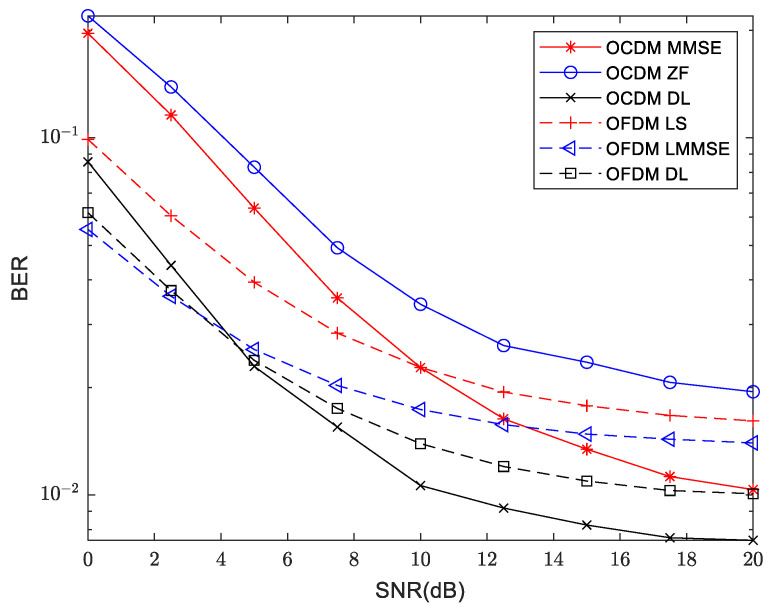
Comparison of BER between traditional and deep learning approaches for OFDM and OCDM at 300 m distance.

**Figure 20:**
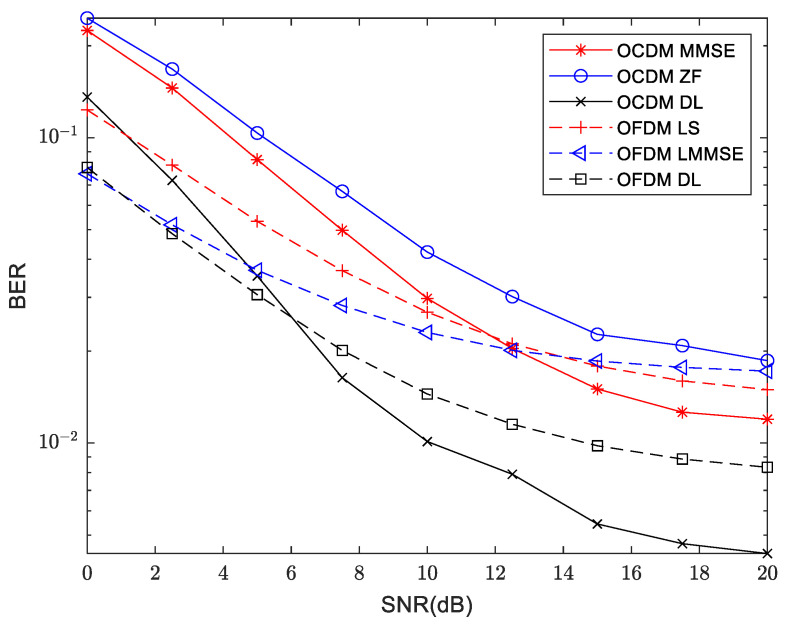
Comparison of BER between traditional and deep learning approaches for OFDM and OCDM at 3000 m distance.

The BER at 300 m and 3000 m are shown in Figure 19 and Figure 20, respectively. It can be seen from the three sets of channel models that the BER performance of OCDM is inferior to that of the OFDM system at low SNR region. However, with increasing SNR, OCDM achieves lower BER compared to the OFDM system. When deep learning is applied to OFDM and OCDM systems, the performance is superior to their respective traditional methods, with more significant improvements observed in the OCDM system. Under medium to high SNR region, OCDM outperforms the OFDM system in terms of BER performance.

## 5. Conclusions

In this paper, VDSR is applied with the shallow water UWA-OCDM communication system, converting the pilot-based channel estimation algorithm into an image super-resolution problem. Additionally, modifications have been made to the VDSR basic framework to handle the CFR matrices and the activation function is replaced by PReLU to ensure that negative numbers can be back propagated and CFR matrix are transformed into dual-channel matrices to account for the correlation between the real and imaginary parts. In order to consider the time-varying characteristics of underwater acoustic communication during the training process, joint SNR training is conducted instead of a traditional network with a single SNR. Experiments show that channel estimation using VDSR in the OCDM system achieves superior noise reduction and interpolation performance compared to traditional estimation methods. The performance of BER is improved by 2.5 dB compared to the MMSE method.

## Figures and Tables

**Table 1:** Parameters of simulator.

Parameter	Value
Number of subcarriers	128
OCDM FT size	128
OFDM FFT size	128
Modulation	QPSK
Type of guard interval	CP
Length of guard interval	32
Channel bandwidth	1280 Hz
Symbol duration	100 ms
Noisy model	White gaussian
Channel model	UWA multipath channels

## Data Availability

The data presented in this study are available upon request from the corresponding author.
